# Withdrawal

**Published:** 1899-09

**Authors:** 


### Withdrawal.

We very much regret that circumstances have occurred which impels the New Jersey State Board of Dental Registration and Examination in Dentistry to withdraw its membership from the National Association of Dental Examiners, and especially is this a matter of regret under the existing circumstances.

It does seem that the National body should have all possible support and co-operation. It has ever been a matter of regret that the State Boards were not more generally represented in this body than they have been. It is to be hoped that circumstances will be so shaped in the near future that this body can again take its place in the National Association and co-operate in the work of that organization. This body has a great influence in the profession, and it is unfortunate for any of its members to withdraw their co-operation. We hope this breach will be healed and the career of this body be more efficacious than ever before.

